# Clinical Features and Impact on One Year Prognosis of Prescribing Low Doses of Direct Oral Anticoagulant Agents in a Middle Eastern Population with Atrial Fibrillation: Analysis from the Jordan Atrial Fibrillation Study

**DOI:** 10.1016/j.ajmo.2023.100058

**Published:** 2023-09-24

**Authors:** Ahmad Alayyat, Munir Zaqqa, Ayman Hammoudeh, Daria Jaarah, Mohammad Bahhour, Mohammed Nawaiseh, Imad Alhaddad

**Affiliations:** a Hamilton Medical Center, Dalton, Ga; b Al Khalidi Hospital and Medical Center, Amman, Jordan; c Istishari Hospital, Amman, Jordan; d Jordanian Royal Medical Services (JRMS), Amman, Jordan; e Jordan Hospital, Amman, Jordan

**Keywords:** Anticoagulation, Direct oral anticoagulant agents, Middle East, Nonvalvular atrial fibrillation, Stroke, Systemic embolism

## Abstract

**Introduction:**

Direct oral anticoagulant agents (DOACs) are indicated for stroke prevention in patients with nonvalvular atrial fibrillation (NVAF). Reduced doses of DOACs are indicated in patients who have renal impairment and according to age and weight criteria. The aim of this study was to investigate the frequency, clinical factors, and impact on 1-year prognosis of underdosing DOACs.

**Methods:**

Data of patients enrolled in the Jordan AF (JoFib) study and who were followed for 1 year was used to compare patients prescribed standard dose with those who were underdosed.

**Results:**

There were 672 patients (76.2%) who were prescribed standard dose and 210 patients (23.8%) who were underdosed. Baseline characteristics were similar between the 2 groups. Factors associated with underdosing were enrollment from an outpatient vs hospital site, moderate- or high-risk HAS-BLED score, an abnormal left ventricular ejection fraction (LVEF <50%), a history of heart failure, or current use of diuretics. At 1 year, the incidence of all-cause mortality was 12.2% in standard dose vs 13.3% in the underdose group (*P* = .82), stroke or systemic embolism was 3.6% in the standard dose vs 3.8% in the underdose group (*P* = .67), and major bleeding was 2.2% in the standard dose vs 3.3% in the underdose group (*P* = .35).

**Conclusions:**

About (25%) of patients were underdosed. Factors associated with underdosing were outpatient (vs hospital) center enrollment, moderate- or high-risk HAS-BLED score, abnormal LVEF (<50%), history of heart failure, and current use of diuretics. There were no significant differences in the incidence of adverse events of mortality and major morbidity at 1-year follow-up between the standard dose and the underdose groups.

## Introduction

Atrial fibrillation (AF) is the most common chronic cardiac arrhythmia, affecting 1%-2% of the general population.^1^ AF predisposes to thrombosis within the left atrium and subsequent embolization and stroke. Anticoagulation with vitamin K antagonists (VKA) was found to significantly reduce this complication.^2^ Direct oral anticoagulant agents (DOACs) are increasingly used in place of warfarin for stroke and systemic embolism (SSE) prevention in patients with nonvalvular AF (NVAF) after the publication of several clinical practice guidelines.^3^ This shift was the result of several advantages of DOACs over VKA that include fewer food and drug interactions and simplified dosing regimens with no need for periodic coagulation monitoring. DOACs are prescribed at fixed doses utilized in clinical studies, but lower doses might be indicated when there is impaired clearance of the drug. Clinical studies have demonstrated DOACs benefit with the use of regular dose and clinically indicated reduced dose according to certain clinical criteria and in the presence of renal function impairment.^4^ For dabigatran standard dose was considered for both 110 mg twice daily and 150 mg twice daily or 75 mg if there was renal impairment.^5^ Rivaroxaban standard dose was 20 mg or 15 mg in the presence of renal dysfunction.^6^ Edoxaban standard dose was 60 mg or 30 mg once daily in the presence of renal dysfunction, body weight ≤ 60 kg, or concomitant use of P-glycoprotein inhibitors.^7^ Apixaban standard dose was 5 mg twice daily or 2.5 mg twice daily if there was renal impairment, an age of ≥80 years, or body weight ≤ 60 kg.^8^ Real-world studies have shown that physicians frequently prescribe reduced doses of DOACs when there is perceived risk of bleeding and in the absence of clear and proven indications (underdosing).^9^ In this study we investigated the frequency, clinical determinants, and impact on 1-year prognosis of underdosing compared to standard dosing in patients with NVAF in a Middle Eastern population enrolled in the Jordan AF (JoFib) study.

## Methods

### Study Population

We analyzed data from the JoFib study which enrolled 2020 consecutive adult patients with AF in 29 hospitals and outpatient clinics in Jordan (May 2019 through October 2020). The study was a prospective one. Follow-up was conducted through phone calls and patient interviews during outpatient clinic visits or hospital admissions. Audits were performed at 6 and 12 months to ensure accurate data collection.

**Figure 1:**
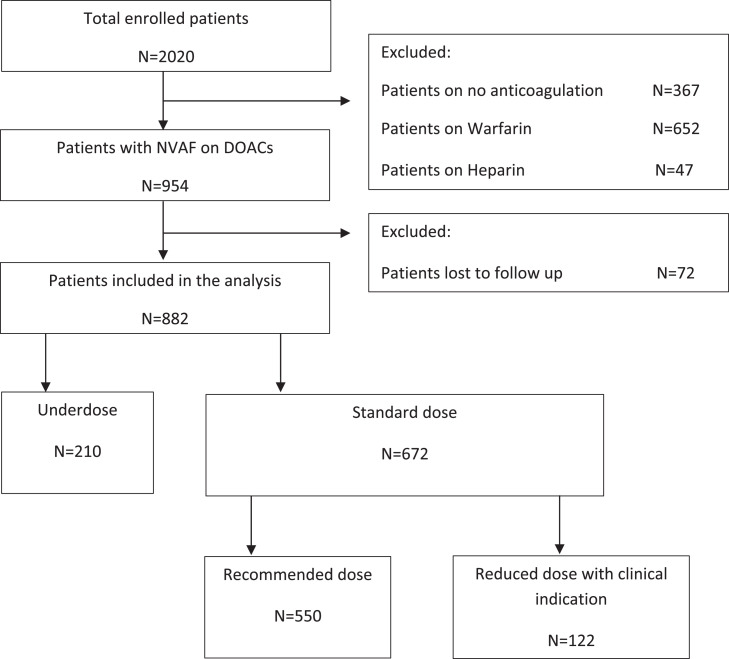
Patients included in the study. DOACs = direct oral anticoagulants; NVAF = nonvalvular atrial fibrillation.

A prespecified endpoint of the study was to evaluate the features and outcome of patients with NVAF who were underdosed. The JoFib study methods and baseline data have been reported previously.^10^ Of the patients initially enrolled, we excluded patients who were not on DOACs and those who were lost to follow-up. Patients prescribed DOACs and completed 1 year of follow-up were included in this analysis (Figure 1).

Clinical data and incidence of 1-year outcome of all-cause mortality, all-cause hospitalization, SSE, and major and clinically relevant nonmajor (CRNM) bleeding events were compared between underdose and standard-dose groups. Patients who had renal impairment (by history or with a creatinine of >1.4 mg/dL), age or weight criteria according to manufacturer recommendations of DOACs, and received reduced dose were included in the standard dose group.

Stroke was diagnosed based on neurologist evaluation according to standard clinical and imaging criteria. Systemic embolism (SE) was diagnosed based on documented clinical, angiographic, intraoperative, or pathological evidence of atheroembolism to an arterial bed of the extremities or abdominal aorta branches. Major bleeding event was defined based on the International Society of Thrombosis and Hemostasis definition and included fatal bleeding, symptomatic bleeding in a critical area or organ (ie, intracranial, intraspinal, intraocular, retroperitoneal, intraarticular or pericardial), and/or bleeding that causes a fall in hemoglobin level of >2 g/dL, or requires >2 units of whole blood or red cells transfusion.^11^ CRNM was defined as a bleeding event that results in hospitalization, requires medical and/or surgical evaluation or intervention, or requires physician-directed change in antithrombotic regimen.^12^

The CHA_2_DS_2_-VASc score was used to assess the thromboembolic risk and the risk for stroke in patients with NVAF. High-risk patients included women with a score of 3 or more and men with a score of 2 or more.^13^ HAS-BLED score was used for bleeding risk assessment.^14^ HAS-BLED patients with a score of 0 point were considered to have a low risk, while patients with ≥1 point were considered to have moderate to high risk for bleeding.

Patients were divided into 2 groups: the standard dose group which included patients who received the recommended dose according to manufacturer criteria and the underdose group which included those who received reduced dose without manufacturer recommendation for such reduced dose. Institutional Review Boards of participating centers approved the study. Each patient signed a written informed consent. The study was registered at clinicatrials.gov (unique identifier number NCT03917992).

### Statistical Analysis

Data analysis was performed using the Statistical Package for Social Sciences software (SPSS) version 25.0. Demographic properties of the patients were analyzed by descriptive statistics. Chi square and independent t-tests were used to determine the differences between the 2 groups among categorical and continuous variables, respectively. Continuous data were expressed as mean ± standard deviation (SD) and categorical data by frequency and percentage. The normality tests conducted for height, weight, BMI, and age revealed that all of these variables exhibited a normal distribution. A *P* value of <.05 was considered statistically significant. Demographic variables assessed included age, body mass index (BMI), sex and past cardiovascular (CV) history, diabetes mellitus (DM), hypertension (HTN), chronic renal failure (CRF), current smoking status, and dyslipidemia and CV medications.

In our study, we included all adverse events that occurred within the patient population, with some patients having more than an event. By including all events, we aimed to capture a comprehensive overview of the safety profile and the frequency of adverse events experienced by the patients.

For outcomes, unadjusted comparison, using chi square test, was performed to test the association of DOAC dose and outcomes through the last follow-up (at 1 year after enrolment). Multivariate models were adjusted for clinically relevant patient characteristics that might explain the association between dosing and outcomes, which included: age, sex, center type (clinic vs hospital), BMI, DM, HTN, current smoking status, dyslipidemia, history of stroke or systemic embolization (SSE), history of heart failure (HF), or coronary artery disease (CAD). A categorical variable for dose category with 2 levels (standard dose and underdose) was included in the model, with patients receiving standard dose treated as the reference group. Odds ratios with 95% confidence intervals (CI) are presented, along with the corresponding *P* value.

In order to study whether the effect of underdosing is confounded by the quality-of-care differences between centers, a separate analysis was conducted to adjust for center clustering using a generalized estimating equations binary logistic regression model. Since there were a total of 18 centers included in the study, some of these centers had a small number of observations. This caused the model to be numerically unstable during the estimation process and to have nonestimable coefficients and odds ratios. To address this issue, we grouped the centers into 2 groups: outpatient or hospital enrollment center. The results of this analysis were found to be similar to the binary logistic regression mentioned above in Table 3.

As the SSE outcome is the main reason for using anticoagulation, we calculated the sample size needed to detect a significant difference between the 2 groups, with an alpha value of 0.05 and a power of 0.8. The sample size was determined based on the expected outcome percentages difference between standard dose and underdose groups, observed in previous studies, which fell within the range of 0.05-0.07. The calculated sample size ranged from 601 to 1131 participants for each group.^15^, ^16^, ^17^

## Results

Figure 1 shows how patients were included and classified in the analysis. Of 2020 patients enrolled in the study, we excluded patients on no anticoagulation (*N* = 367), patients on warfarin (*N* = 652), and patients on heparin (*N* = 47). Out of 954 patients who were on DOACs, 72 patients (7.5%) were lost to follow-up, to end up with 882 patients (mean age of 70.1 ± 11.7 years) included in this analysis. Of those, 672 (76.2%) were included in the standard dose and 210 (23.8%) in the underdose group. Table 1 shows the baseline clinical and demographic features of both groups. The underdose group was more likely to have a moderate- or high-risk HAS-BLED score compared to the standard dose group (96.2% vs 87.4%, *P* < .001). The frequency of abnormal left ventricular ejection fraction (LVEF) or a history of HF was more common in the underdose group compared to the standard dose group (28.1% vs 20.3%, *P* = .02, and 28.6% vs 21.0%, *P* = .02). Diuretics utilization was more frequent in the underdose compared to the standard dose group (48.1% vs 38.2%, *P* = .01). The results showed a significant difference in center-type distribution between the standard dose group, with 49.7% from hospitals and 50.3% from clinics, and the underdose group, with 36.7% from hospitals and 63.3% from clinics (*P* = .001).

**Table 1:** Demographics and Medical History of the Included Patients.

Features	Standard dose group *N* = 672	Underdose group *N* = 210	*P* value
Male	313 (46.6%)	84 (40.0%)	.09
Age, mean ± SD (Years)	69.7 ± 12.3	71.1 ± 9.631	.10
Center type			.001
Hospital	334 (49.7%)	77 (36.7%)	
Clinic	338 (50.3%)	133 (63.3%)	
Height (cm)	167.3 ± 7.8	166.3 ± 7.9	.10
Weight, mean ± SD (kg)	82.2 ± 15.9	81.3 ± 16.9	.50
BMI, mean ± SD (kg/m^2^)	29.3 ± 5.4	29.5 ± 6.1	.75
BMI ≥25 mg/m^2^	491 (76.7%)	147 (75.0%)	.62
CHA_2_DS_2_-VASc score			.14
Low risk	17 (2.5% (	2 (1.0%)	
Moderate risk	54 (8.0%)	11 (5.2%)	
High risk	601 (89.4%)	197 (93.8%)	
HAS-BLED score			<.001
Low risk	85 (12.6%)	8 (3.8%)	
Moderate or high risk	587 (87.4%)	202 (96.2%)	
HTN	527 (78.4%)	163 (77.6%)	.81
DM	290 (43.2%)	87 (41.4%)	.66
Current smoking	84 (12.5%)	25 (11.9%)	.82
Dyslipidemia	335 (49.9%)	109 (51.9%)	.60
History of SSE	116 (17.3%)	36 (17.1%)	.97
History of HF	141 (21.0%)	60 (28.6%)	.02
History of CAD	81 (12.1%)	30 (14.3%)	.40
History of thyroid disease	72 (10.7%)	22 (10.5%)	.92
History of active cancer	26 (3.9%)	9 (4.3%)	.79
LVEF			.02
Normal (≥50%)	499 (79.7%)	138 (71.9%)	
Abnormal (<50%)	127 (20.3%)	54 (28.1%)	
Medications			
Antiplatelet agents	245 (36.5%)	77 (36.7%)	.96
Beta-blockers	537 (79.9%)	164 (78.1%)	.57
Amiodarone	174 (25.9%)	60 (28.6%)	.44
Calcium channel blockers	69 (10.3%)	19 (9.0%)	.61
Digoxin	80 (11.9%)	28 (13.3%)	.58
RAAS inhibitor	290 (43.2%)	103 (49.0%)	.13
Diuretics	257 (38.2%)	101 (48.1%)	.01

BMI = body mass index; CAD = coronary artery disease; DM = diabetes mellitus; HF = heart failure; HTN = hypertension; LVEF = left ventricle ejection fraction; SSE = stroke or systemic embolization; RAAS = renin-angiotensin-aldosterone system.

Table 2 provides information on the proportion of each drug that is considered underdosed within its respective group. For dabigatran, 6 cases (5.8%) were in the underdose group. For rivaroxaban and apixaban, 138 (31.0%) and 66 (20.1%) patients were in the underdose group, respectively. None of the patients who used edoxaban were underdosed, and only 1 (100%) patient was given the standard dose. These results provide insights into the proportion of underused drugs, specifically rivaroxaban, apixaban, and other DOACs, within different underdosed groups.

**Table 2:** Rates of DOACs Prescription, in the Underdose and Standard Dosing Group.

Drug	Underdose group 210 (23.8%)	Standard dose group 672 (76.2%)	
		Recommended dose 550 (62.4%)	Reduced dose with an indication 122 (13.8%)
Dabigatran	6 (5.8%)	98 (94.2%)	0 (0%)
Rivaroxaban	138 (31.2%)	293 (66.1%)	12 (2.7%)
Apixaban	66 (19.8%)	158 (47.3%)	110 (32.9%)
Edoxaban	0 (0%)	1 (100%)	0 (0%)

DOAC = direct oral anticoagulant.

Clinical outcomes at 1 year of follow-up, stratified by DOACs doses and adjusted logistic regression models, are shown in Table 3. Frequencies of adverse events were not statistically different between underdosed and standard dosed patients. Moreover, after adjusting for center clustering using generalized estimating effect models, underdosing did not demonstrate any statistically significant effect on adverse events (Table 4).

**Table 3:** Multivariate Logistic Regression: Unadjusted and Adjusted Association Between Appropriateness of DOAC Dosing and Clinical Outcomes.

Factor	Level	Number of events	Unadjusted		Adjusted*	
			OR (95% CI)	*P* value	OR (95% CI)	*P* value
All-cause mortality	Standard dosing	86 (12.8%)	1.00		1.00	
	Underdosing	28 (13.3%)	1.04 (0.66, 1.65)	.84	1.01 (0.58, 1.70)	.99
All-cause hospitalization	Standard dosing	199 (29.6%)	1.00		1.00	
	Underdosing	62 (29.5%)	0.99 (0.70, 1.40)	.98	1.01 (0.69, 1.48)	.97
ACS hospitalization	Standard dosing	12 (1.8%)	1.00		1.00	
	Underdosing	2 (1.0%)	0.52 (0.11, 2.38)	.39	0.27 (0.03, 2.20)	.22
Cardiovascular hospitalization	Standard dosing	83 (12.4%)	1.00		1.00	
	Underdosing	27 (12.9%)	1.04 (0.62, 1.66)	.93	0.93 (0.56, 1.55)	.79
Noncardiovascular hospitalization	Standard dosing	80 (11.9%)	1.00		1.00	
	Underdosing	26 (12.4%)	1.04 (0.65, 1.67)	.85	1.10 (0.66, 1.80)	.70
SSE	Standard dosing	24 (3.6%)	1.00		1.00	
	Underdosing	8 (3.8%)	1.06 (0.47, 2.41)	.87	1.21 (0.52, 2.89)	.66
ACS	Standard dosing	13 (1.9%)	1.00		1.00	
	Underdosing	2 (1.0%)	0.487 (0.10, 2.17)	.33	0.23 (0.03, 1.80)	.16
Major bleeding	Standard dosing	15 (2.2%)	1.00		1.00	
	Underdosing	6 (2.9%)	1.28 (0.50, 3.36)	.60	1.21 (0.44, 3.28)	.70
Nonmajor clinically significant bleeding	Standard dosing	37 (5.5%)	1.00		1.00	
	Underdosing	19 (9.0%)	1.63 (0.80, 2.75)	.20	1.51 (0.81, 2.81)	.19

ACS = acute coronary syndrome; DOAC = direct oral anticoagulant; SSE = stroke or systemic embolization.

⁎ Adjusted for age, BMI, sex, smoking status, center type, history of DM, HTN, and dyslipidemia, developed CAD, HF, stroke, or SE, prescription of any of the antiplatelet medications.

**Table 4:** Binary Logistic Regression Using the Generalized Estimating Equations Model: The Association Between Appropriateness of DOAC Dosing and Clinical Outcomes After Adjusting for Clustering of Centers.

Factor	Level	Adjusted*	
		OR (95% CI)	*P* value
All-cause mortality	Standard dosing	1.00	
	Underdosing	1.04 (0.65, 1.64)	.879
All-cause hospitalization	Standard dosing	1.00	
	Underdosing	0.98 (0.70, 1.39)	.923
ACS hospitalization	Standard dosing	1.00	
	Underdosing	0.51 (0.11, 2.32)	.383
Cardiovascular hospitalization	Standard dosing	1.00	
	Underdosing	0.95 (0.59, 1.53)	.829
Noncardiovascular hospitalization	Standard dosing	1.00	
	Underdosing	1.06 (0.65, 1.71)	.827
SSE	Standard dosing	1.00	
	Underdosing	1.00 (0.44, 2.27)	.991
ACS	Standard dosing	1.00	
	Underdosing	0.46 (0.10, 2.10)	.317
Major bleeding	Standard dosing	1.00	
	Underdosing	1.43 (0.56, 3.66)	.456
Nonmajor clinically significant bleeding	Standard dosing	1.00	
	Underdosing	1.61 (0.12, 2.92)	.310

ACS = acute coronary syndrome; DOAC = direct oral anticoagulant; SSE = stroke or systemic embolization.

⁎ Adjusted for center clustering.

## Discussion

One of the main advantages of DOACs over VKA is prescribing a fixed dose that can be reduced according to specific clinical conditions without the need for blood monitoring. However, reduced doses of DOACs are frequently used in conditions other than what was specified by manufacturer labeling. This has the potential of having less bleeding, but more thromboembolic complications.

In the current study underdosing DOACs was in 24% of the studied population. This percentage is in the range of what was found in previous studies.^18^, ^19^, ^20^ Factors which were found to be associated with underdosing included moderate- or high-risk HAS-BLED score, an abnormal (<50%) LVEF, or a history of HF and current use of diuretics. Uni- and multivariate analysis showed no decrease in bleeding nor an increase in adverse events of thrombosis, hospitalization, or overall mortality at 1-year follow-up.

Although a simple fixed dose may result in better compliance, a wide range of factors may affect metabolism and may lead to adverse events. Accounting for factors that affect metabolism is therefore important when prescribing medications with a narrow therapeutic index such as DOACs. Clinical judgment may result in underdosing based on factors that affect metabolism other than those considered in the labeling. Finding these factors may help us further in refining the use of DOACs.

Among patients who were prescribed reduced dose with clinical indication, apixaban was the primary medication, accounting for 90% of this group. The reason behind such finding is thought to be that the criteria for dose reduction of apixaban are the broadest.

A similar outcome in the 2 groups of patients in this study may be due to the factors found to be different between underdose and regular groups. Enrolment from clinic was associated with using an underdose of DOAC in comparison to enrollment from a hospital. This might be related to the experience of enrolling physicians from these centers and greater concern for the risk of bleeding in comparison to the risk of stroke. Another factor may be due to the decision being made individually in the outpatient setting while being multidisciplinary in the hospital. Higher HAS-BLED score is expected to be associated with factors related to higher bleeding which may be the driver behind underdosing DOACs. Other factors found in this study were history of HF, abnormal LVEF, and use of diuretics which are related to low cardiac output. Liver congestion and subsequent effect on metabolism and production of coagulation factors may result in increased bleeding risk.^21^ Although major clinical studies did not show increased bleeding risk with DOACs in HF patients when compared to warfarin, real-world data showed bleeding to be a significant problem.^22^, ^23^, ^24^

We found a similar risk of bleeding, thrombosis, hospitalization, and overall mortality between the 2 groups, even after adjusting to center clustering. Such similar outcome is comparable to what was reported in other studies.^19^^,^^20^^,^^25^ One study reported increased CV hospitalization in underdosed patients, but this could be a reflection of higher morbidity of the underdose group requiring more hospitalizations rather than the effect of the medication itself.^18^

The reasons for underdosing DOACs need to be studied further to find if there is a merit behind the decision or otherwise educate the physicians on adhering to the recommended dose. Additional prospective studies are needed to further characterize patients who may benefit from underdosing to improve clinical outcomes and improve our standing on this subject.

This study has few limitations. The observational design of the study is inherently associated with the potential bias of residual confounding clinical factors, collection of patients’ data, and recall of events. Study investigators were urged to recruit consecutive patients from different sectors of the local health care system. The major CV events evaluated at 1 year were hard endpoints, such as death, SSE, and major bleeding, which are unlikely to be affected by recall issues. Percent lost to follow-up (7.5%) is consistent with what is found in studies but still may hamper the discriminatory ability of some analyses and the generalizability of the results. Another limitation is that the relatively small number of patients who were included (882 of a total of 2020 patients enrolled in the whole study) might make the study underpowered to detect any clinical outcome significant differences. For example, the sample size for the underdose group (*n* = 210) was smaller than the recommended sample size range of 601-1131, as indicated by the SSE outcome sample size calculation, potentially limiting our ability to detect a difference even if one actually existed and increasing the risk of type II error. Also, the study did not analyze variation in results based on centers. Other limitations in this study that can be considered in future studies are that results were not adjusted for clustering of events at the patient level and the study did not include patient adherence. Despite these limitations, this study represents an important contribution to the contemporary knowledge of baseline features and 1-year outcome in a Middle Eastern population with AF who were prescribed lower than recommended doses of DOACs.

## Conclusions

One in four patients in this real-world AF population was prescribed lower than recommended DOACs doses. Patients in the underdose group had moderate or high HAS-BLED score and history of HF or abnormal ejection fraction and were on diuretic treatment. There was no significant difference in clinical outcomes or adverse events between the underdose and standard dose groups. Further studies are needed to evaluate the factors that are associated with using reduced DOAC doses and to prospectively study the impact of this practice on the incidence of major bleeding events and thromboembolic outcomes.

## Declaration of Competing Interest

The authors declare that they have no known competing financial interests or personal relationships that could have appeared to influence the work reported in this paper.
